# A Non-Symmetric Reconstruction Technique for Transcriptionally-Active Viral Assemblies

**DOI:** 10.13188/2474-1914.1000004

**Published:** 2015-08-17

**Authors:** Amina Rahimi, A. Cameron Varano, Andrew C. Demmert, Linda A. Melanson, Sarah M. McDonald, Deborah F. Kelly

**Affiliations:** 1 Virginia Tech Carilion Research Institute, Roanoke, VA, USA; 2 Virginia Tech Carilion School of Medicine, Roanoke, VA, USA; 3 Life Sciences Division, Gatan, Inc, Pleasanton, CA, USA; 4 Department of Biomedical Sciences and Pathology, Virginia-Maryland College of Veterinary Medicine, Virginia Tech, Blacksburg, VA, USA; 5 Department of Biological Sciences, Virginia Tech, Blacksburg, VA, USA

**Keywords:** Rotavirus, Double-layered particles, Transcription, Affinity capture, Cryo-electron microscopy

## Abstract

The molecular mechanisms by which RNA viruses coordinate their transcriptional activities are not fully understood. For rotavirus, an important pediatric gastroenteric pathogen, transcription occurs within a double-layered particle that encloses the viral genome. To date, there remains very little structural information available for actively-transcribing rotavirus double-layered particles, which could provide new insights for antiviral development. To improve our vision of these viral assemblies, we developed a new combinatorial strategy that utilizes currently available high-resolution image processing tools. First, we employed a 3D classification routine that allowed us to sort transcriptionally-active rotavirus assemblies on the basis of their internal density. Next, we implemented an additional 3D refinement procedure using the most active class of DLPs. For comparison, the refined structures were computed in parallel by (1) enforcing icosahedral symmetry, and by (2) using no symmetry operators. Comparing the resulting structures, we were able to visualize the continuum that exists between viral capsid proteins and the viral RNA for the first time.

## Introduction

**Figure 1:**
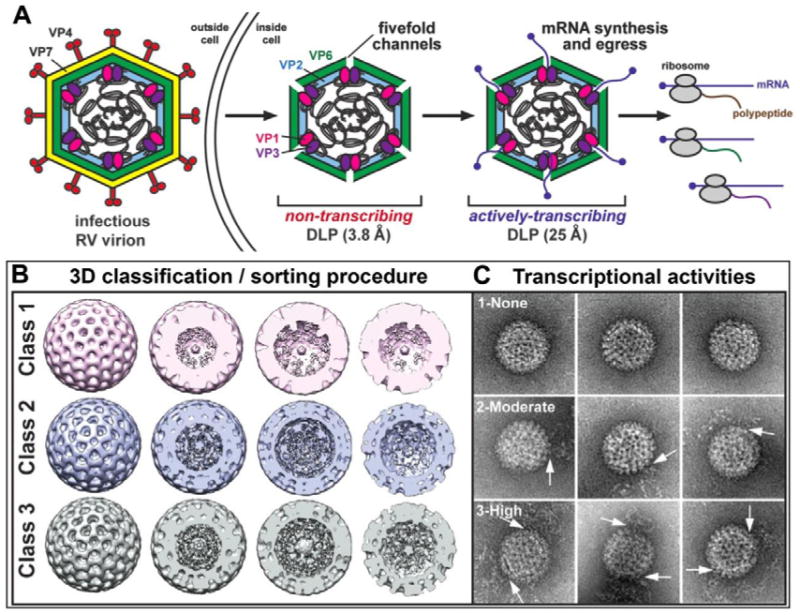
Schematic to indicate viral processes that can occur during host-cell infection and initial structural insights of active RV assemblies **(A)** The infectious RV virion sheds its outer most protein layer (VP4-VP7) as it enters the host cell forming a double-layered particle (DLP). VP1 and VP3 proteins coordinate the production of mature mRNA transcripts of RV genome segments. The mRNA transcripts are then extruded into the cytoplasm of the host cell. Host ribosomes are responsible for translating the viral mRNAs into viral proteins. **(B)** Cryo-EM 3D reconstructions of DLPs (Class 1 - 3) having different levels of internal density. The diameter of each reconstruction is 80 nm. **(C)** Representative images of negatively stain DLPs that comprise similar Class 1, 2, or 3 reconstructions. The transcriptional activities of the particles (none, moderate, or high) were interpreted based upon the amount of surrounding mRNA. White arrows point to single-stranded mRNAs. These results suggest that Class 3 particles, which are robustly transcribing, have the most ordered internal density. The width of each panel is 200 nm. Figures in panels (B) and (C) were adapted from Kam et al. [10].

RNA viruses are ubiquitous in nature and represent some of the most severe pathogens known to mankind (e.g. influenza A virus, Ebola virus, poliovirus, etc.). Rotaviruses (RVs) are non-enveloped, eleven-segmented, double-stranded RNA viruses that can cause life-threatening gastroenteritis in children. RVs express their genes as single-stranded, messenger RNA (mRNA) molecules early in infection through the process of transcription [1]. RV transcription occurs within intact, subviral double-layered particles (DLPs) within the cytoplasm of the host cell [2]. Transcriptionally competent DLPs (Figure 1A) are formed when the outermost VP7-VP4 layer of the mature, triple-layered RV virions (TLP) are shed during cell entry [3]. DLPs primarily contain viral genomic RNA that is encapsulated by a protective protein layer (VP6). Also found within DLPs are core shell proteins (VP2), viral polymerases (VP1), and mRNA capping proteins (VP3) that coordinate the production of mature mRNA transcripts [4]. In the cytoplasm, viral transcripts are recognized by host cell ribosomes and act as functional templates for protein synthesis (Figure 1A) [5]. Given the importance of RV transcripts during the viral replication cycle, it is expected that abrogation of their synthesis by targeted antiviral will effectively prevent RV-induced disease. Still, the molecular mechanisms by which RV DLPs are able to function as such exquisite mRNA-producing nanomachines are not fully understood, consequently hampering the design of pharmacological inhibitors.

Three-dimensional (3D) structures of non-transcribing RV DLPs have been determined to high resolution (3.8 Å) using cryo-electron microscopy (cryo-EM) or X-ray crystallography [6,7]. Not only have these structures revealed atomic-level details of VP6 and VP2, they have also provided insight into the location of viral polymerase complexes (comprised of VP1 and VP3) within the particle [8]. Specifically, in the maps of the inactive DLP, a hub of density is detected just below each 5-fold axis, which is partially attributed to VP1/VP3 complexes. Unfortunately, the only available 3D structures of actively transcribing DLPs were determined >15 years ago at lower resolution (25 Å) [5,9]. These active DLP structures show mRNA exiting the DLP through VP2-VP6, but they do not reveal any detailed features of the particle.

Recent cryo-EM structural assessments performed by our laboratories revealed the unexpected finding that transcriptionally-active DLPs exhibited varying degrees of disorder in their external VP6 capsids [10]. Additionally, the DLPs with the most disordered external density contained more internally ordered features within the 3D structure. Upon inspecting the individual DLP assemblies that represented the active structures, we also found greater quantities of mRNA surrounded the particles (Figures 1B and 1C, Class 3). Complimentary to this result, particles exhibiting externally ordered capsids observed in the same cryo-EM images correlated with very little internally ordered density; and the particles that constituted this structure were surrounded by very little mRNA in the EM images (Figures 1B and 1C, Class 1). Yet another 3D structure exhibited some external disorder and some internally ordered features, having a modest degree of mRNA in the area surrounding the particles (Figures 1B and 1C, Class 2) [10]. Here, we build upon these intriguing findings to gain a deeper understanding of protein and genomic RNA arrangements within transcriptionally-active DLPs. Specifically, we address the following questions: 1) in computing 3D structures of active DLPs, how does the use of icosahedral symmetry during the refinement procedures influence the resulting density map; and 2) what structural information can we gain about active viral assemblies without assuming icosahedral symmetry during refinement procedures?

## Materials and Methods

### DLP preparation

Rotavirus (strain SA11-4F) DLPs were prepared as described in our previous work [10]. Transcription activation reactions (25 μl each) were performed in eppendorf tubes and each mixture contained the following components: 1 μg DLPs prepared in 100 mM Tris-HCl pH 7.5, 6 mM MgAc, 4 mM DTT, 2 mM each of ATP, GTP, CTP, UTP, and 1 μl RNasin (Promega Corp, Madison, WI). The reactions mixtures were incubated for ∼30 minutes at 37 °C. After the incubation period, aliquots of the transcription reactions (3 μl each) were used for Affinity Capture experiments.

### Affinity-capture experiments

EM Affinity grids containing Nickel-nitrilotriacetic acid (Ni-NTA) coatings were prepared using holey carbon grids (C-flat-2/1 grids; Protochips, Inc.) as previously described [10]. The Ni-NTA functionalized layers were comprised of 25% Ni-NTA lipids and 75% 1,2-dilauryl-phosphatidylcholine (DLPC) filler lipids (Avanti Polar Lipids). Protein adaptors were sequentially added to the Ni-NTA coated grids. We first added aliquots (3 μl) of His-tagged Protein A (0.01 mg/ml) (Abcam) in buffer solution containing 50 mM HEPES, pH 7.5, 150 mM NaCl, 10 mM MgCl_2_ and 10 mM CaCl_2_. The Protein A solution was incubated for 1 minute on each grid and the excess solution was blotted away with filter paper. Next, 3 μl aliquots of VP6-specific guinea pig polyclonal anti sera (#53963) (0.01 mg/ml) contained in the same HEPES buffer solution were added to Protein A-decorated grids. After a 1 minute incubation step, the excess solution was gently removed using a Hamilton syringe. Finally, the transcriptionally active DLPs (2 μl aliquots of 0.1 mg/ml) were added to the antibody-decorated grids for a 2 minute incubation. Frozen-hydrated specimens were prepared by plunge-freezing the grids into liquid ethane slurry using a Gatan Cryoplunge™ 3 equipped with Gentle Blot capabilities (Gatan, Inc.) while employing a one-sided blotting routine for approximately 8 seconds.

### Electron microscopy

Frozen-hydrated specimens were transferred to a Gatan 626 cryo holder (Gatan, Inc.) and maintained under liquid nitrogen until transferred into the TEM. Specimens were imaged using a FEI Tecnai Spirit BioTwin TEM (FEI, Co, Hillsboro, OR) equipped with a LaB_6_ filament and operated at an acceleration voltage of 120 kV under low-dose conditions (∼5 electrons/ Å^2^). Images of transcriptionally-active DLPs were recorded on a FEI Eagle 2k HS CCD camera with a pixel size of 30 μm using a defocus range from -1.0 μm to -3.0 μm at a nominal magnification of 60,000× for a final sampling of 5 Å/pixel at the specimen level.

### Image processing and 3D refinement

Images of transcriptionally-active DLPs were recorded as described above and individual particles were selected from the images using automated routines in the PARTICLE software package (http://www.image-analysis.net/EM). Prior to particle selection, the raw images were inverted, normalized and CTF-corrected using standard routines in PARTICLE. The final image stack containing 2769 particles was exported in MRC format for 3D reconstruction and refinement calculations in the RELION software package [11]. Within the RELION package, we used a reference map for the DLP structure [7] that was available from the website of the Grigorieff laboratory. Implementing the RELION program, 3D classification routines identified 3 distinct classes while enforcing icosahedral symmetry through 25 cycles of refinement. The particles that comprised the 3D structure and displayed the most disordered exterior exhibited well-defined density within the internal particle. These particles were then subjected to 10 subsequent rounds of refinement while enforcing either C1 or I1 symmetry, and while dividing the particles into two equal halves. The resulting 3D structures were resolved to ∼15 Å as independently verified using the RMEASURE program [12]. Overall parameters for the global reconstruction routines included a pixel size of 5 Å, a reference model low-pass filtered to 50 Å, and a regularization parameter of T=4 over an angular search space of 7.5 degrees.

## Results and Discussion

### Images of transcriptionally-active DLPs reveal non-symmetric features

Improving our understanding of protein-RNA interactions can provide important new insights to dissect infection at the molecular level. The dynamic manner in which these interactions occur remains unclear, especially in the case of viral mRNA production. To expand upon our previous findings of transcriptionally-active RV DLPs, we implemented a new computing protocol to better visualize the dynamic nature of these complexes. We collected images of frozen-hydrated transcriptionally-active DLPs that were tethered to C-flat holey carbon Affinity Grids (Protochips, Inc.). These grids were decorated with adaptor molecules including IgG antibodies against the VP6 capsid protein (please see Methods section). EM images of the DLP specimens were acquired using a FEI Tecnai Spirit BioTwin TEM (FEI, Co, Hillsboro, OR) equipped with a LaB_6_ filament and operated at an acceleration voltage of 120 kV under low-dose conditions (∼5 electrons/ Å^2^).

**Figure 2:**
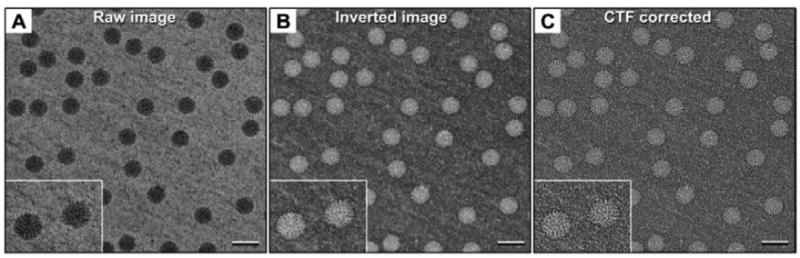
EM images of actively transcribing DLPs show variable features among the individual viral assemblies **(A)** Representative EM image of transcriptionally active DLPs in ice. The image contrast was inverted and normalized **(B)** then CTF-corrected **(C)** using standard procedures in the PARTICLE software package, providing refined images for 3D reconstruction. Insets highlight individual DLPs with surrounding mRNA. Scale bar is 100 nm.

The raw images (Figure 2A) revealed DLP assemblies having a diameter between 80-90 nm, some of which were associated with RNA strands, consistent with our previous observations [10,13]. A number of particles in the images displayed strongly symmetric features although these attributes varied among the population. To further understand the structural variation in the DLPs, we processed the images for downstream reconstruction procedures. Using the PARTICLE software package (http://www.image-analysis.net/EM), we inverted and normalized each image (Figure 2B) then implemented standard routines for CTF correction (Figure 2C). Individual DLPs were selected from the corrected images using the automated processes implemented in PARTICLE as previously described [10]. The resulting image stack containing 2769 individual particle images was imported into the RELION software package for 3D reconstruction and refinement routines.

### 3D classification routines can sort DLPs on the basis of structural variability

The EM images clearly indicated that particle variability existed within the active DLP population. Hence, computing a variety of 3D structures may better represent the inherent mobility present among the diverse population of complexes. We used a standard 3D classification routine within the RELION software package to test for the number of statistically significant 3D structures present in our stack of DLP images. Based on Bayesian inference and utilizing a filtered starting model (50 Å) of the DLP structure [7], RELION identified three distinct structures present in our images after 25 cycles of refinement. This observation is consistent with our previous structures (Figure 1B) that were computed by applying icosahedral symmetry operators (I1 space group) during the 3D classification process [10]. Again, we noted that the structures having distinctly ordered internal features correlated with the greatest degree of proximal mRNA.

Overall, the 3D classification routine reproducibly distinguished various structures among the active particles. However, considering the fact that not all DLPs displayed perfect symmetry in the images, we investigated whether applying the icosahedral symmetry operator to the active DLPs obscured information about protein-RNA arrangements. We performed additional refinement calculations using only the particles contained within the presumed most active class (Figure 1, Class 3). These DLPs contained the strongest internal features and had greater quantities of nearby mRNA strands in the images. We calculated new 3D structures of these assemblies using the RELION software package, with and without the use of symmetry operators (I1 vs C1 space groups). In each case, we used the same reconstruction parameters, varying only the symmetry operators during the calculations. We implemented 10 refinement cycles in RELION while dividing the data into two halves for direct comparison.

### 3D reconstructions reveal a continuum of features that represent integrated protein-RNA density

**Figure 3:**
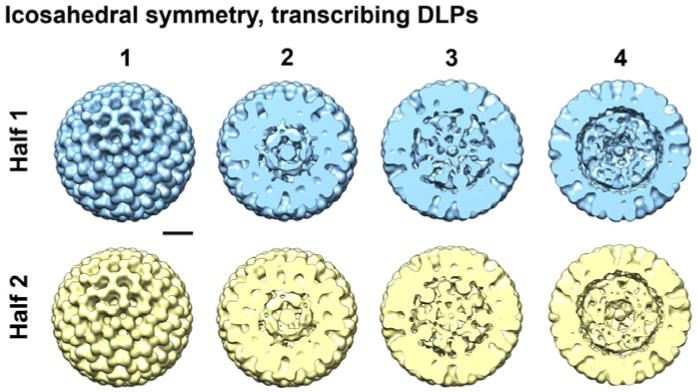
Icosahedral reconstructions of active DLPs reveal internal structural features Particles that comprised the Class 3 structure shown in Figure 1B were refined using the RELION software package while enforcing icosahedral symmetry. The images were equally divided into two halves (blue and yellow) during the refinement procedure. Contoured sections are shown at 10 nm intervals through the density ending at the midsection of the particle. 3D reconstructions are ∼80 nm in diameter. Scale bar is 20 nm.

Upon examining the resulting reconstructions generated from applying icosahedral symmetry, we visualized strong internal density along the 5-fold axis in contoured sections through the EM structures (Figure 3). Protein subunits arranged distinctively along the 5-fold axis have been previously identified as VP1 and VP3 in non-transcribing DLPs. Our new symmetrized density maps resolved to ∼15 Å resolution also revealed strong globular density at this position and is consistent with the work of Lawton and colleagues [5,9]. By examining through the sections of the density map, we also visualized features that collectively defined the protein-RNA contribution to the particle integrity. This information was missing from the original analyses of non-transcribing DLPs, which were focused on defining high-resolution features in the VP6 protein capsid [7].

**Figure 4:**
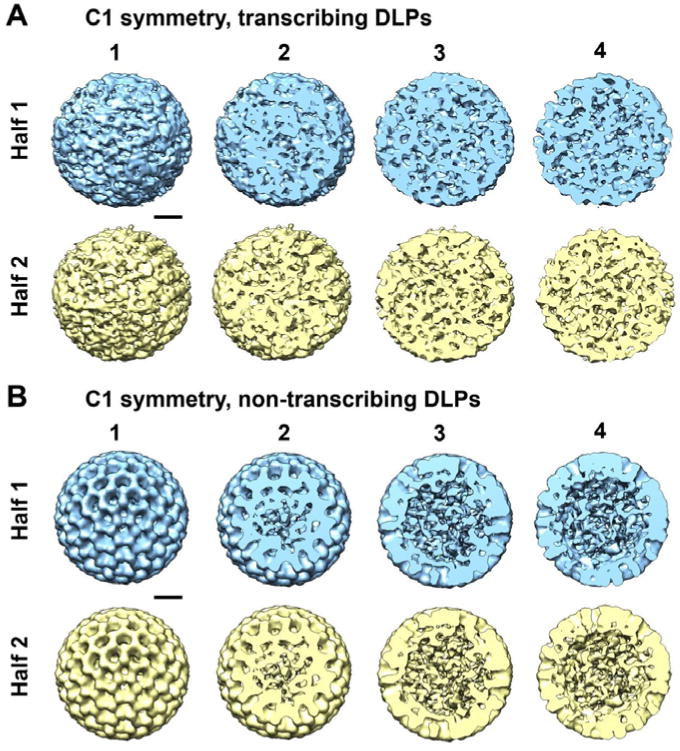
Non-symmetric reconstructions of active DLPs reveal structural variability in a continuous manner throughout the density maps **(A)** Particles that comprised the Class 3 structure shown in Figure 1B were refined using the RELION software package in the C1 space group. The images were equally divided into two halves (blue and yellow) during the refinement procedure. Contoured sections are shown at 10 nm intervals through the density ending at the midsection of the particle. 3D reconstructions are ∼80 nm in diameter. **(B)** As an internal control, we calculated EM reconstructions using the C1 space group for inactive DLPs. The resulting structures were highly symmetric as expected, considering the strong symmetry elements that were visible within the individual particles that constituted this population. Scale bar is 20 nm.

Upon examining the 3D density maps calculated from the same images of active DLPs, but without imposing icosahedral symmetry, we found very different results (Figure 4A). The overall structures appeared to be strongly assymetric, but showed a continuum of density throughout the maps. In comparing the same cross-sections of the non-symmetrized reconstructions with those of the icosahedral structures, we found that the inherently disordered C1 structures did not show distinct 5-fold channels, nor did they provide a clear view of individually distinct subunits. However, when we consider the fact that the active DLP assemblies that comprised the reconstructions were similarly lacking in strong symmetry elements, it seems reasonable that the more dynamic reconstructions may also provide a realistic view of the most active DLPs structures.

As a control experiment, additional 3D structures were calculated in the C1 space group for non-transcribing particles contained within the same image stack (Figure 4B). For the reconstructions of non-transcribing assemblies that were calculated without imposing icosahedral operators, we still observed strong symmetry elements within the resulting structures as expected for the inactive assemblies. As an external control, we also examined cryo-EM images of non-transcribing DLPs prepared in buffer solution but lacking the required elements to induce transcriptional activity. EM structures computed in the C1 space group for these inactive assemblies indicated a statistically single population having features consistent with the reconstructions shown in Figure 4B.

In comparing our results to the high-resolution structures of the transcriptionally-active subviral particle of cypo virus, a member of the same viral family as RV (i.e., *Reoviridae*), we found an essential commonality- a series of conformational changes must occur in the viral capsid during mRNA synthesis, capping, and egress [14]. It remains unknown whether this process is being driven by the structural mobility of the internal RNA genome or by fluid protein rearrangements in response to the changing RNA landscape during transcription.

Collectively, the experimental results presented here complement the multi scale molecular dynamics simulations of Miao and Ortoleva [15]. Their studies showed that structural transitions in viral capsids begin locally then propagate across the continuum of the outer protein layer. Their findings also support the idea of non symmetric intermediate states among ordered, icosahedral viral assemblies. In particular, they suggested that the use of symmetry elements alone does not reveal the full regime of viral structural transitions [15].

Additional work by Vlijmen and Karplus demonstrated the existence of structural variability among viral transition states using normal mode analysis while applying icosahedral symmetry [16]. Although both native and altered states of viral capsids displayed symmetrical elements, the transitional pathway to achieve these variable conformations was not ordered. In fact, less than 2% of viral capsid motions were considered to be symmetrical in nature, indicating that asymmetrical transition states are more likely to dominate atomic fluctuations [16].

Finally, studies performed by Brooks et al. described the need to examine viral capsid reconstructions with and without enforcing symmetry elements [17,18]. Their reasoning for these considerations draws from the fact that for part of the viral lifespan, protein capsids can assume an icosahedral symmetric state. However, non symmetric intermediates must also exist due to the elastic properties of the capsid constituents and changes in morphology permitted by buckling transition theory [17,18].

Based on these theoretical frameworks, and considering the continuum of density visualized in the more disordered structures presented here, it seems feasible that the movements within the DLP cores are influenced by RNA-induced mobility in cooperation with internal protein subunits. As such, the transitional movements in the VP6 capsid are likely to be asymmetric due to the natural fluctuations needed to accommodate RNA synthesis and extrusion. We expect similar movements may occur in other pathogenic RNA viruses, and that our strategies can be broadly utilized to provide new insights for structure-based drug design. Overall, we anticipate future work involving *in situ* TEM analysis of transcriptionally-active viral assemblies within a liquid environment may reveal additional information to delineate real-time mechanisms within these exquisite nanomachines.

## Abbreviations

RV: Rotavirus
DLPs: Double-Layered Particles
mRNA: messenger RNA
EM: Electron Microscopy
Ni-NTA: Nickel-Nitrilotriacetic acid
nm: Nanometers
3D: Three-dimensional

## References

[1] McDonald SM (2013). RNA synthetic mechanisms employed by diverse families of RNA viruses. Wiley Interdiscip Rev RNA.

[2] Jayaram H, Estes MK, Prasad BV (2004). Emerging themes in rotavirus cell entry, genome organization, transcription and replication. Virus Res.

[3] Trask SD, McDonald SM, Patton JT (2012). Structural insights into the coupling of virion assembly and rotavirus replication. Nat Rev Microbiol.

[4] Prasad BV, Chiu W (1994). Structure of rotavirus. Curr Top Microbiol Immunol.

[5] Lawton JA, Estes MK, Prasad BV (1997). Three-dimensional visualization of mRNA release from actively transcribing rotavirus particles. Nat Struct Biol.

[6] McClain B, Settembre E, Temple BR, Bellamy AR, Harrison SC (2010). X-ray crystal structure of the rotavirus inner capsid particle at 3.8 Å resolution. J Mol Biol.

[7] Zhang X, Settembre E, Xu C, Dormitzer PR, Bellamy R (2008). Near-atomic resolution using electron cryomicroscopy and single-particle reconstruction. Proc Natl Acad Sci U S A.

[8] Estrozi LF, Settembre EC, Goret G, McClain B, Zhang X (2013). Location of the dsRNA-dependent polymerase, VP1, in rotavirus particles. J Mol Biol.

[9] Lawton JA, Estes MK, Prasad BV (1999). Comparative structural analysis of transcriptionally competent and incompetent rotavirus-antibody complexes. Proc Natl Acad Sci U S A.

[10] Kam JA, Demmert AC, Tanner JR, McDonald SM, Kelly DF (2014). Structural dynamics of viral nanomachines. Technology.

[11] Scheres SH (2012). A Bayesian view on cryo-EM structure determination. J Mol Biol.

[12] Sousa D, Grigorieff N (2007). Ab initio resolution measurement for single particle structures. J Struct Biol.

[13] Tanner JR, Demmert AC, Dukes MJ, Melanson LA, McDonald SM (2013). Cryo-SiN- an alternative substrate to visualize active viral assemblies. J Anal Mol Tech.

[14] Yang CW, Jia G, Liu HR, Zhang K, Liu GQ (2012). Cryo-EM structure of a transcribing cypovirus. Proc Natl Acad Sci U S A.

[15] Miao Y, Ortoleva PJ (2010). Viral structural transition mechanisms revealed by multiscale molecular dynamics/order parameter extrapolation simulation. Biopolymers.

[16] van Vlijmen HW, Karplus M (2005). Normal mode calculations of icosahedral viruses with full dihedral flexibility by use of molecular symmetry. J Mol Biol.

[17] May ER, Brooks CL (2012). On the morphology of viral capsids: elastic properties and buckling transitions. J Phys Chem B.

[18] May ER, Feng J, Brooks CL (2012). Exploring the symmetry and mechanism of virus capsid maturation via an ensemble of pathways. Biophys J.

